# The Elastic Modulus and Damage Stress–Strain Model of Polypropylene Fiber and Nano Clay Modified Lime Treated Soil under Axial Load

**DOI:** 10.3390/polym14132606

**Published:** 2022-06-27

**Authors:** Zhichao Wang, Weiqing Zhang, Ping Jiang, Cuihong Li

**Affiliations:** School of Civil Engineering, Shaoxing University, Shaoxing 312000, China; 21020859083@usx.edu.cn (Z.W.); zhangweiqingusx@163.com (W.Z.); jiangping@usx.edu.cn (P.J.)

**Keywords:** lime treated soil, polypropylene fiber, nano clay, mechanical properties, damage model

## Abstract

Using polypropylene fiber (PPF) and nano clay modified lime treated soil (LS), the static and dynamic properties of fiber modified lime treated soil (FLS), nano clay modified lime treated soil (NLS), and fiber nano clay composite modified lime treated soil (NFLS) were studied. Through the unconfined compressive strength (UCS) test and dynamic triaxial test of FLS, NLS, and NFLS, the static and dynamic elastic modulus characteristics at 7 day curing age were explored, and the damage stress–strain model was established. The results show that: (1) Polypropylene fiber and nano clay can significantly enhance the mechanical properties of NFLS. Nano clay can promote the reaction between lime and soil to produce calcium silicate hydrate (C-S-H) and calcium aluminate hydrate (C-A-H), thus improving the strength of NFLS, and UCS can be increased by up to 103%. Polypropylene fiber can enhance the ductility of NFLS and increase the residual ductility strength, and the residual strength can be increased by 827%. (2) Nano clay can enhance the static and dynamic elastic modulus of modified lime treated soil. The static and dynamic elastic modulus of NLS, FLS, and NFLS are linear with the change of polypropylene fiber and nano clay content. The static and dynamic elastic modulus of NLS, FLS, and NFLS are linear, exponential, and logarithmic, respectively. (3) The mesoscopic random damage model can characterize the stress–strain relationship of NFLS. Polypropylene fiber and nano clay can improve the ductility and strength of modified LS, and the composite addition of polypropylene fiber and nano clay can improve the ability of modified LS to resist damage.

## 1. Introduction

Due to the demand of economic and urbanization development, the quality requirements of road infrastructure construction are increasing year by year. Using lime treated construction waste soil for road base is an effective measure to reduce construction waste. At the same time, it can replace sand, stone, and other materials, so as to reduce the irreversible damage to the ecological environment caused by mountain excavation and river excavation. The traffic load is mainly axial load. Due to the increase of road load, higher requirements are put forward for the mechanical properties of road base. Therefore, it is necessary to deeply explore the properties of lime treated soil and the main technical means to improve its mechanical properties.

Liu et al. [1] proved that after adding lime into the soil, the soil properties change, which can improve its unconfined compressive strength (UCS) to meet the actual engineering needs. Lime can not only significantly improve the UCS of the improved soil and reduce the expansion and contraction of the soil, but also react with the pozzolanic ash to form cementitious materials, which is an excellent material for stabilizing the soil [2,3]. Lemaire et al. [4] conducted UCS and X-ray diffraction (XRD) tests on lime treated soil. The results show that lime can make the soil form a “honeycomb” structure and significantly improve the mechanical properties of the soil. Lime treated soil can improve the mechanical properties of soil to a certain extent, but its brittle failure mode will affect the strength and durability of road base. Therefore, further study of the mechanical properties of LS modified by adding polypropylene fiber and nano clay is required [5,6]. Jiang et al. [7] studied the interfacial action and energy dissipation of polypropylene fiber and glass fiber modified iron tailings through single fiber drawing test, and established a quantitative relationship model between dissipated energy, effective fiber length, and freeze–thaw cycle. The UCS, CBR, and TCT tests of polypropylene fiber modified soil show that the addition of polypropylene fiber will improve the strength, ductility, and residual strength of soil [8,9]. With the progress of science and technology, nano clay can significantly improve the mechanical properties of soil due to its unique physical and chemical properties, and has begun to become a new stable material in the field of construction engineering [10]. Niu et al. [11] studied the mechanical properties, durability, shrinkage, hydration, and microstructure of cement-based materials modified by nano clay. The results show that an appropriate amount of nano clay is beneficial to improve the properties of cement-based materials. Gao et al. [12] conducted triaxial undrained and SEM tests on nano MgO modified soil, and found that nano MgO can effectively improve the strength and cohesion of engineering waste silt and reduce the porosity of soil. Many scholars have studied the modification characteristics of nano materials, and the results show that nano materials can improve the compressive strength of LS and improve the compression characteristics of LS [13,14,15]. Lu et al. [16] studied the effects of moisture content, compactness, cyclic deviatoric stress, and confining pressure on the dynamic elastic modulus of lime treated expansive soil by using dynamic triaxial test, and verified the applicability of UT Austin model. Zhao et al. [17] measured the stress–strain curve of solidified soft soil under different lime content and confining pressure through dynamic triaxial test, and analyzed the relationship between dynamic and static strength and the influence of lime content on dynamic strength. Therefore, fiber materials and nano materials play a positive role in the mechanical properties of modified LS.

Fiber materials and nano materials have good engineering practical significance for modified LS. Many scholars have deeply studied the stress–strain model of modified materials. Zhou et al. [18] discussed the relationship between the failure mechanism, deformation characteristics, and strength of lime treated expansive soil, and constructed a nonlinear elastic model. Poinard et al. [19] revealed the main differences between damage and failure mechanisms by studying the meso damage mechanism of concrete under hydrostatic pressure and triaxial pressure, using X-ray computed tomography instrument. Jiang et al. [20] established UCS and STS prediction models of NFLS by studying the effects of nano clay and polypropylene fiber on the unconfined compressive strength and splitting performance of LS, taking the content of nano clay, pore volume, and lime volume as independent variables. Wang et al. [21] studied the stress relaxation of lime treated unsaturated soil according to a series of triaxial tests, and established a structural model considering time effect to characterize the cumulative damage of LS.

Polypropylene fiber and nano clay are used to modify LS. The mechanical properties of FLS, NLS, and NFLS were studied through UCS test and dynamic triaxial test. Based on the meso random damage theory, the stress–strain damage relationship of NFLS is established, which provides a theoretical reference for the application of modified LS in road engineering.

## 2. Materials and Methods

### 2.1. Materials

The raw materials used in the test were soil, quicklime powder, polypropylene fiber, and nano clay. The soil was from the foundation pit construction site in Shaoxing City, Zhejiang Province, China. The soil is grayish brown, and its main physical and mechanical properties are shown in Table 1. The quicklime powder was produced by Jiangxi Xinyu Liangliang Trading Co., Ltd., Xinyu City, Jiangxi Province, China, with CaO content of 88% and purity of 98%. The polypropylene fiber was a bundle monofilament, which was produced by Shaoxing Fiber High Tech Co., Ltd., Shaoxing City, Zhejiang Province, China, with a diameter of 18–46 μm and length of 6 mm, which has the characteristics of light weight and good stability, and can significantly improve the ductility and bending resistance of soil. The nano clay used was produced by Hubei Jinxi montmorillonite Co., Ltd., Wuhan City, Hubei Province, China, the density is 0.45 g/cm^3^, 99.9% of nano clay can pass the 200 mesh screen, and the content of montmorillonite is 96–98%, which is beige powder.

### 2.2. Test Scheme

LS was modified by nano clay, polypropylene fiber, composite nano clay and polypropylene fiber. The static and dynamic properties of the modified LS were studied by UCS test and dynamic triaxial test. The content of lime was 6%, and, on this basis, nano clay with the content of 0%, 2%, 4%, and 6% and polypropylene fiber with the content of 0%, 0.25%, 0.5%, and 0.75% was added respectively. The content of lime, nano clay, and polypropylene fiber was the mass ratio of dry soil. The mechanical properties of NFLS were investigated by adding 2%, 4%, and 6% nano clay in the optimal FLS and 0.25%, 0.5%, and 0.75% polypropylene fiber in the optimal NLS [20].

**Figure 1:**
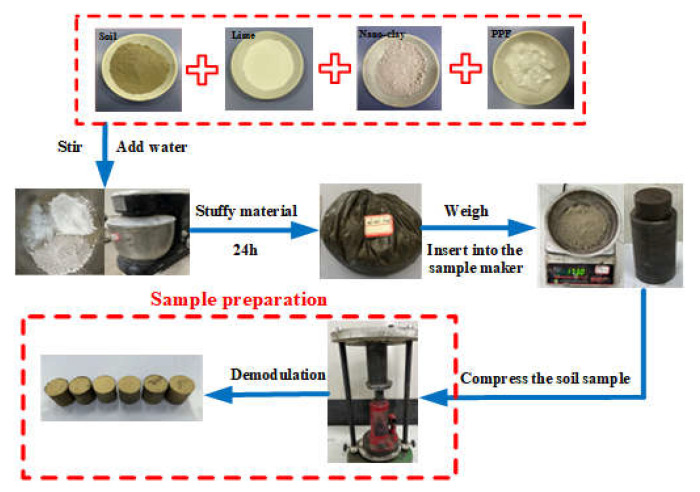
Sample preparation process.

According to the Chinese test code for Inorganic Binder Stabilized Materials in Highway Engineering [22], the undisturbed soil of the required soil sample is crushed and dried in a 105 °C oven. After crushing, a 2 mm sieve is used to screen the soil. According to the test scheme, after weighing the corresponding quality of dry soil, quicklime, nano clay, and polypropylene fiber with an electronic scale, add water and stir evenly according to the optimal moisture content to make each admixture disperse and not agglomerate. Put the uniformly stirred aggregate into the sealing stuffs of plastic bags for 24 h, and make samples by static compaction, as shown in Figure 1. After demoulding, it should be cured for 7 days. In the first 6 days, it should be cured in a standard curing box with a temperature of 20 ± 2 °C and a relative humidity of more than 95%. On the last day, the sample should be taken out of the curing box and soaked in water at 20 ± 2 °C for curing. The water surface is approximately 2.5 cm above the sample. The test scheme is shown in Table 2.

**Figure 2:**
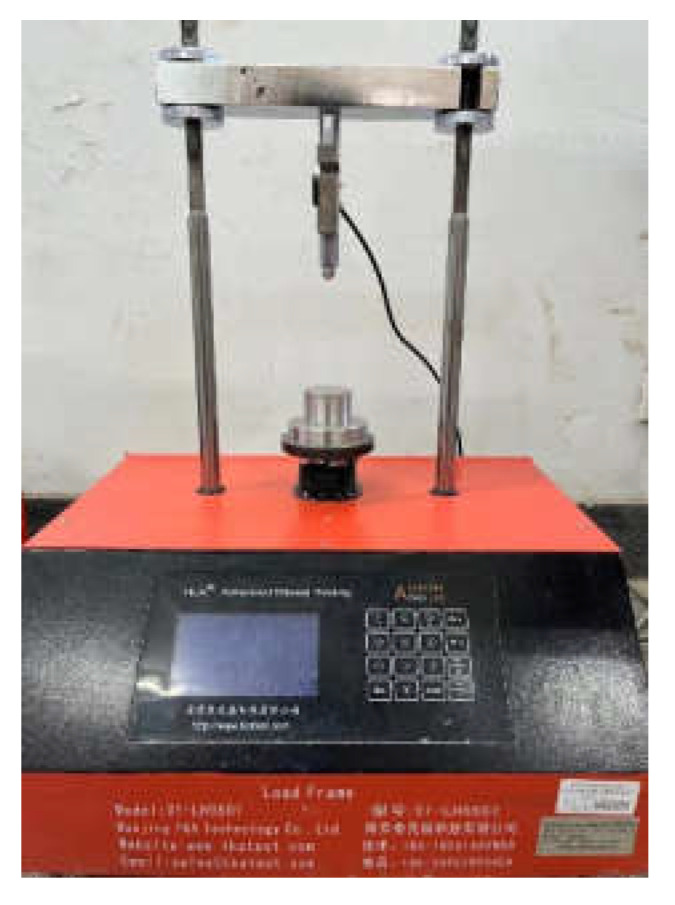
UCS test system.

The UCS test sample is a cylinder with a diameter of 50 mm and a height of 50 mm. After curing, TKA-WCY-1F automatic unconfined compressive strength tester is selected for testing, as shown in Figure 2. Referring to the Chinese code for Highway Geotechnical Test [23], the loading rate of the instrument was 1 mm/min, and the stress–strain curve of the sample is obtained through the computer data acquisition system.

**Figure 3:**
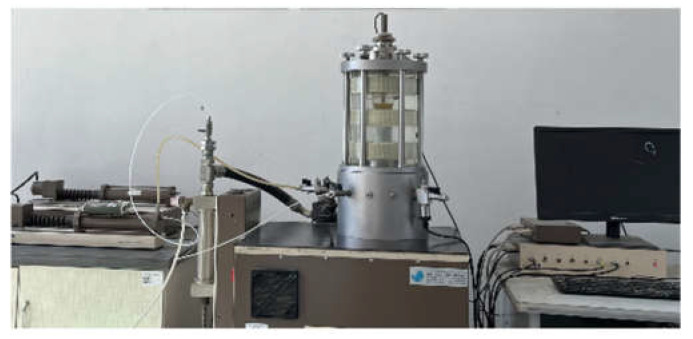
Dynamic triaxial test loading system.

Because the traffic load has a long-term load impact on the road base, the dynamic elastic modulus of NFLS is studied by dynamic triaxial test to deeply explore the long-term traffic load bearing capacity of modified LS. After curing, GDS servo motor control system is selected for dynamic triaxial test. The size of the dynamic triaxial test sample is a cylinder with a diameter of 39.1 mm and a height of 80 mm. Because the surrounding earth pressure has little impact on the road base, the confining pressure is 0 kPa, and the displacement control method is used to vibrate for 50 times. The test is carried out with a sine wave with a frequency of 1 Hz and an amplitude of 0.1 mm. Data acquisition is carried out through microcomputer system, as shown in Figure 3.

For SEM test, a Nissan high-low vacuum scanning electron microscope (JSM-6360LV, JEOL, Tokyo, Japan) was used. The electro-adhesive was evenly spread on the aluminum tray, and then the powder of the sample to be tested was glued to the electro-adhesive. After the required test sample was glued, a layer of platinum film was uniformly sprayed on the surface of the sample. After confirming uniform spraying, the aluminum tray was put into the electron microscope for SEM test.

FT-IR was tested by KBr compression method. The instrument used in the experiment was the Fourier Infrared spectrometer (NEXUS, Thermo Nicolet Corporation, American, Waltham, MA, USA), with a wavelength range of 4000–400 cm^−1^. The test sample was ground into powder and then added into potassium bromide powder. Grinding continued so that the sample powder and potassium bromide powder was fully mixed and ground into a fine powder. Then the powder was pressed into sheets by KBr method and put into the instrument for infrared spectrum analysis.

### 2.3. Stress–Strain Damage Model

According to the random damage mechanics of concrete, Li et al. [24,25] abstracted the concrete sample as a micro spring random damage system from the meso point of view, so as to establish the random damage constitutive model of concrete. According to the meso random damage theory, the damage stress–strain relationship of materials under compression is shown in Equation (1).

(1) $$\sigma =\left(1-D\right)E\varepsilon$$

where *E* is the elastic modulus, *D* is the damage variable, and the mean and variance of *D* are shown in Equations (2) and (3), respectively.

(2) $$\mu_{D}=\frac{\left(1-\beta \right)\mu_{D_{S}}}{1-\beta \mu_{D_{S}}}$$

(3) $$V_{D}{}^{2}=\frac{\left(1-\beta \right)\mu_{D_{S}}}{{\left(1-\beta \right)}^{2}}\left(2\int_{0}^{1}\left(1-\eta \right)F_{S}\left(\gamma ,\gamma ,\eta \right)d\eta -\mu_{D_{S}}\right)$$

The meso random damage model is mainly related to parameters *λ*, *ζ*, and *ω* [26,27]. *E* can be obtained from the dynamic triaxial test of NFLS, and then the parameters of *λ*, *ζ*, and *ω* can be identified according to the UCS test results, so as to establish the stress–strain damage model of NFLS.

## 3. Results and Discussion

### 3.1. Static Characteristics

#### 3.1.1. UCS Test Results

UCS and residual strength are important mechanical indexes for the study of NFLS. The peak value of stress–strain curve is taken as the UCS, and the stress corresponding to peak strain plus 5% is selected as the residual strength. The UCS test results of NFLS are shown in Table 3.

**Figure 4:**
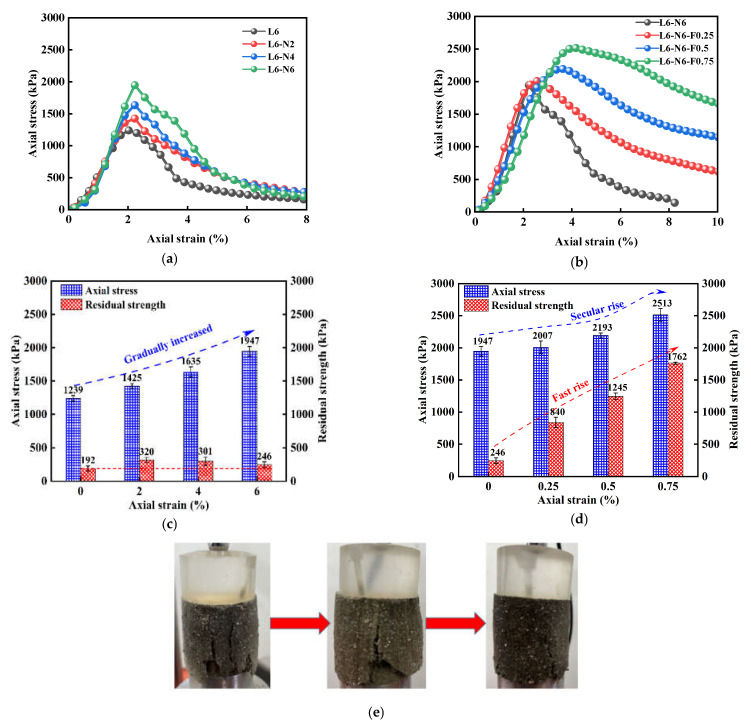
Effect of PF on the statics of NFLS: (**a**) stress–strain curve of NLS; (**b**) stress–strain curve of NFLS (N = 6%); (**c**) UCS and residual strength of NLS; (**d**) UCS and residual strength of NFLS (N = 6%); and (**e**) the variation of cracks with the content of polypropylene fiber under the same strain.

Figure 4a,b show the stress–strain curve of NFLS with nano clay content N = 6%. The stress–strain curve of NFLS is a softening curve, that is, the stress first increases to the stress peak with strain, then decreases, and then tends to be gentle. Figure 4c,d show the UCS and residual strength of NFLS with nano clay content N = 6%. The increase of nano clay and fiber content can significantly improve the UCS of LS. The content of nano clay increases from 0% to 6%. The UCS of NFLS increases with the increase of nano clay content. When the content of nano clay is 6%, the UCS of NLS is 1947 kPa and the relative growth rate of UCS is 57%. When the optimal nano clay content is 6%, the UCS of NFLS increases with the increase of polypropylene fiber content, and when the fiber content is 0.75%, the UCS of NFLS is 2513 kPa, the growth rate relative to NLS is 29%, and the growth rate of UCS is the fastest. It shows that the strength of NFLS can be significantly improved by adding nano clay and fiber, and the optimal contents of nano clay and polypropylene fiber are 6% and 0.75% respectively. The residual strength of NLS increases first and then decreases with the increase of nano clay content, which is due to the increase of brittleness of NLS and the decrease of residual strength. When the content of nano clay is 6%, the residual strength of NFLS increases linearly with the increase of the content of polypropylene fiber. This is because the fiber can slow down the development of failure cracks of NFLS. With the increase of the content of fiber, the cracks develop more slowly and the bearing capacity is greater, indicating that polypropylene fiber can significantly enhance the ductility of NFLS and slow down its brittle failure. The interfacial force generated between the polypropylene fiber and soil can play a better role in the bonding of cracks. As shown in Figure 4e, under the same strain, with the increase of polypropylene fiber content, the fibers emerging from the crack of the sample increase significantly, the pull effect is significant, and the crack development slows down [28,29].

**Figure 5:**
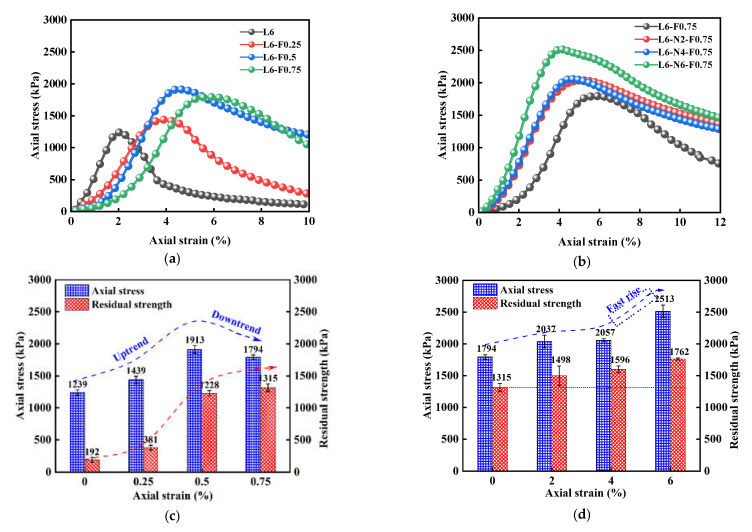
Effect of nano clay on the statics of NFLS: (**a**) stress–strain curve of FLS; (**b**) stress–strain curve of NFLS (F = 0.75%); (**c**) UCS and residual strength of FLS; (**d**) UCS and residual strength of NFLS (F = 0.75%).

**Figure 6:**
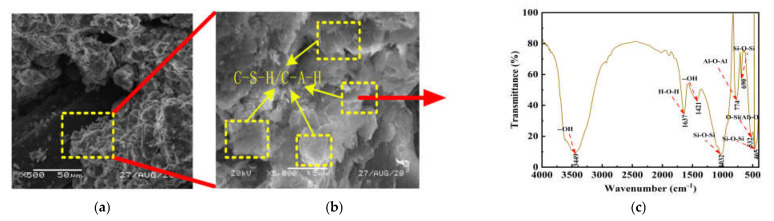
Microscopic analysis graphs of NFLS: (**a**) 500−fold SEM; (**b**) 5000−fold SEM; (**c**) FT−IR.

Figure 5a,b shows the stress–strain curve of FLS and NFLS with fiber content F = 0.75%, and Figure 5c,d shows the UCS and residual strength diagram of NFLS with fiber content F = 0.75%. With the increase of polypropylene fiber content, the UCS of FLS first increases and then decreases. When the polypropylene fiber content is 0.75%, the UCS of FLS shows a downward trend, which is due to the overhead phenomenon when the network structure is formed between the soil mass due to the excessive fiber content, and the relative compactness of the soil mass decreases, leading to a decrease in UCS growth. When the fiber content is 0.75%, the UCS of NFLS increases with the increase of nano clay content, and when the nano clay content is 6%, the UCS of NFLS increases the fastest. This is because nano clay can fill the pores between LS particles and make its internal structure more dense. Secondly, nano clay can promote the further reaction of LS, and on the other hand, it can have pozzolanic reaction with LS [30,31,32,33]. The volcanic ash reaction generates a sheet structure, as shown in Figure 6a. The sheet structure is cementitious substances such as hydrated calcium silicate C-S-H and hydrated calcium aluminate C-A-H, as shown in Figure 6b,c. The generated cementitious material can fill the pores between the LS skeleton and make the LS particles agglomerate better. At the same time, the interface between fiber and soil particles is closer, the interfacial friction is greater, the damage degree of external force to the sample is reduced, and the strength of NFLS is further increased. However, the residual strength of NFLS increases slowly with the increase of nano clay content, indicating that polypropylene fiber contributes more to the residual strength of NFLS than nano clay.

The above phenomena show that nano clay mainly provides strength for NFLS, but will enhance the brittle failure of LS. Polypropylene fiber can enhance the ductility of NFLS and improve its residual strength.

#### 3.1.2. Variation Law of Static Elastic Modulus

**Figure 7:**
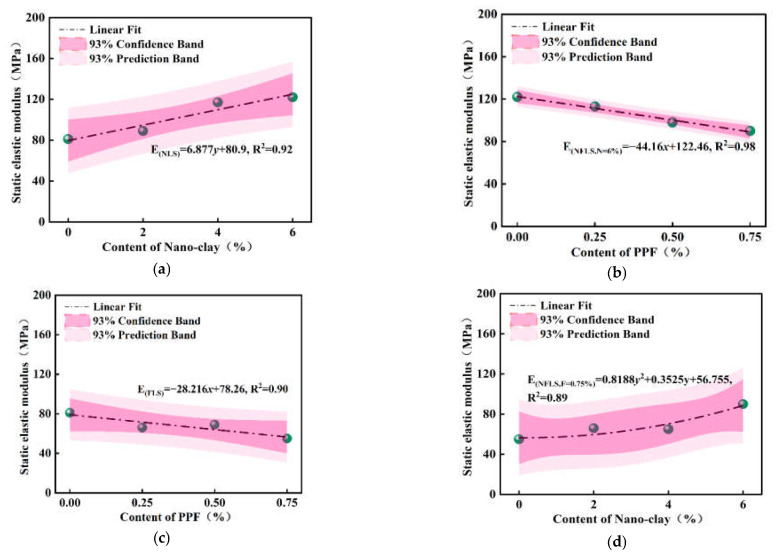
Static elastic modulus: (**a**) static elastic modulus curve of NLS; (**b**) static elastic modulus curve of NFLS (N = 6%); (**c**) static elastic modulus curve of FLS; (**d**) static elastic modulus curve of NFLS (F = 0.75%).

**Figure 8:**
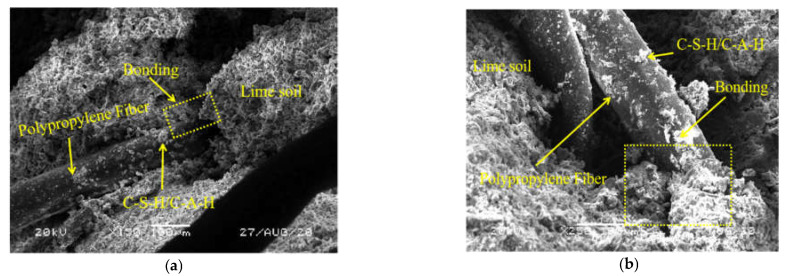
Microcosmic diagram of the interface between PPF and lime-soil: (**a**) 150-fold SEM; (**b**) 250-fold SEM.

The static elastic modulus is the ratio of stress to strain in the elastic deformation stage under compression. Through the unconfined compressive strength test data, the stress–strain curve of NFLS in the elastic stage under compression is fitted, and the straight-line slope is the static elastic modulus. Figure 7a–d shows the variation curve of static elastic modulus under different content of nano clay and polypropylene fiber. Figure 7a shows the variation law of the static elastic modulus of NLS with the increase of nano clay content. When the content of nano clay is 6%, the static elastic modulus of NLS increases to 122 MPa, and the growth rate is 51% higher than that of LS, indicating that the addition of nano clay can improve the static elastic modulus of LS and enhance its deformation resistance. Figure 7b shows that when the content of nano clay is 6%, the static elastic modulus of NFLS decreases with the increase of the content of polypropylene fiber, because the stress of NFLS in the elastic stage decreases with the growth rate of strain. In Figure 7c, it can be found that the content of polypropylene fiber increases from 0.25% to 0.75%, and the static elastic modulus of FLS first increases and then decreases. When the content of polypropylene fiber is 0.5%, the elastic modulus of FLS is 69 MPa, while when the content of polypropylene increases by 0.75%, the static elastic modulus of FLS decreases to 55 MPa, indicating that on the one hand, this is due to the large amount of C-S-H and C-A-H produced by lime soil reaction attached to the surface of polypropylene fiber, so polypropylene fiber can enhance the adhesion of the interface between soil and fiber and make the samples bond together better, as shown in Figure 8. On the other hand, with the increase of the content of polypropylene fiber, the porosity between soil particles and fiber may increase, resulting in the decrease of its static elastic modulus. Figure 7d shows that when the content of polypropylene fiber is 0.75%, the static elastic modulus of NFLS first increases, then decreases, and finally significantly increases with the increase of nano clay content. When the content of nano clay is 6%, the static elastic modulus of NFLS is significantly increased to 90 MPa. This is because nano clay can fill the pores between fiber and soil, enhance the stiffness of NFLS, and reduce elastic deformation.

The relationship is fitted between the static elastic modulus and the content of nano clay and polypropylene fiber of NLS, FLS, and NFLS. The fitting formulas are shown in Equations (4)–(7).

(4) $$E_{\left(\mathrm{NLS}\right)}=6.877y+80.9,R^{2}=0.92$$

(5) $$E_{\left(\mathrm{NFLS},\;N=6\%\right)}=-44.16x+122.46,R^{2}=0.98$$

(6) $$E_{\left(\mathrm{FLS}\right)}=-28.216x+78.26,R^{2}=0.90$$

(7) $$E_{\left(\mathrm{NFLS},F=0.75\%\right)}=0.8188y^{2}+0.3525y+56.755,R^{2}=0.89$$

where *E* is the static elastic modulus (MPa); *x* is the content of polypropylene fiber (%); and *y* is the content of nano clay (%).

### 3.2. Dynamic Characteristics

#### 3.2.1. Hysteretic Curve

**Figure 9:**
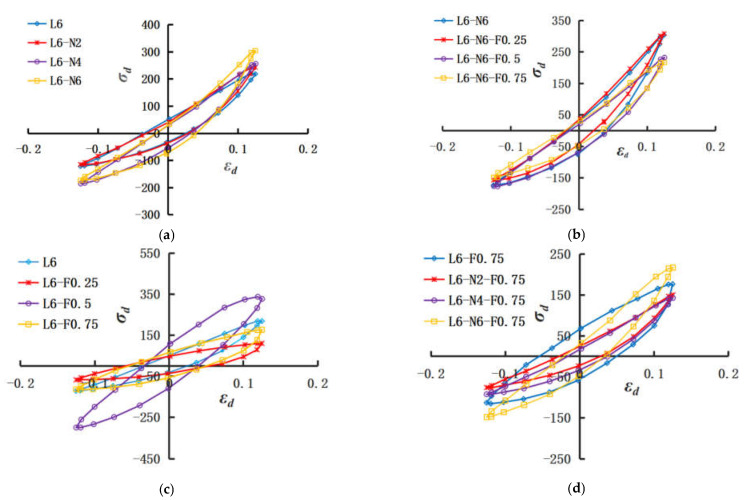
Dynamic stress–strain hysteresis curve: (**a**) change of NLS with nano clay content; (**b**) changes of NFLS (N= 6%) with PPF content; (**c**) changes of FLS with PPF content; (**d**) change of NFLS (F = 0.75%) with nano clay content.

The dynamic elastic modulus of the material can be calculated from the slope of the hysteretic curve to characterize the stiffness of the soil [34]. Figure 9a–d shows the variation law of dynamic stress–strain hysteretic curves of NLS, FLS, and NFLS.

Figure 9a,b shows that the hysteresis curves of NLS gradually approaches the stress axis with the increase of nano clay content. When the content of nano clay is 6%, different content of polypropylene fiber has a significant effect on the hysteretic curve. With the increase of polypropylene fiber content, the angle between hysteretic curve and stress axis increases. The dynamic elastic modulus of NFLS decreases with the increase of polypropylene fiber content. Figure 9c,d shows that the hysteresis curves of FLS fluctuates with the increase of polypropylene fiber content and its area and angle with the stress axis. The FLS area is the largest and the included angle with the stress axis is the smallest when the content of polypropylene fiber is 0.5%. When the content of polypropylene fiber is 0.75%, the inclination angles of hysteresis curves increase gradually with the content of nano clay.

#### 3.2.2. Dynamic Elastic Modulus

**Figure 10:**
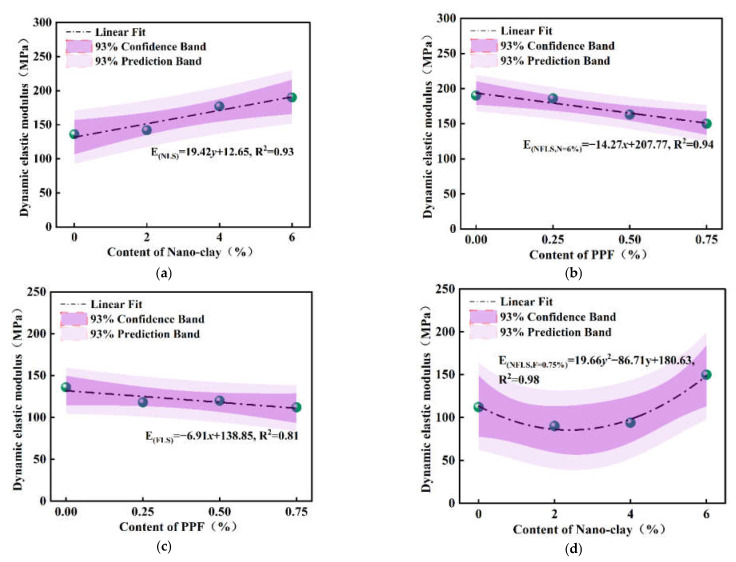
Dynamic elastic modulus: (**a**) dynamic elastic modulus curve of NLS; (**b**) dynamic elastic modulus curve of NFLS (N = 6%); (**c**) dynamic elastic modulus curve of FLS; (**d**) dynamic elastic modulus curve of NFLS (F = 0.75%).

According to the hysteretic curves, the dynamic elastic modulus of NFLS with different nano clay and polypropylene fiber content can be obtained according to Equation (8), as shown in Figure 10.

(8) $$E_{d}=\frac{\sigma_{d}}{\varepsilon_{d}}$$

Figure 10a,b shows that the dynamic elastic modulus of NLS increases with the increase of nano clay content. The nano clay content increases from 0% to 6%, the dynamic elastic modulus of NLS increases to 189 MPa, and nano clay can significantly enhance the elastic modulus of LS. When the content of nano clay is 6%, the dynamic elastic modulus of NFLS decreases with the increase of the content of polypropylene fiber. Nano clay can enhance the dynamic elastic modulus of LS because nano clay can produce pozzolanic reaction by modifying LS to produce more cementitious materials and make its structure more compact. When the sample is pressed and does not break, the strain produced is very small, so the force on the fiber can be decomposed into a compressive force and a flexural force, which has almost no effect on the sample and mainly plays a role of reinforcement [35,36,37].

Figure 10c,d shows that the dynamic elastic modulus of FLS first decreases, then increases and then decreases with the increase of polypropylene fiber content. When the polypropylene fiber content is 0.5%, the dynamic elastic modulus of FLS is 120 MPa, while when the polypropylene fiber content increases to 0.75%, the dynamic elastic modulus decreases to 112 MPa. When the polypropylene fiber content is large, it is easy to agglomerate and interweave, reducing the compactness of soil. The capillary action of fiber will lead to soil erosion and reduce the dynamic elastic modulus of LS. When the content of polypropylene fiber is certain, the dynamic elastic modulus of NFLS decreases first and then increases significantly with the increase in the content of nano clay. When the content of nano clay is 6%, the dynamic elastic modulus of NFLS is 150 MPa.

The relationship between dynamic elastic modulus of NLS, FLS, and NFLS and content of nano clay and polypropylene fiber was fitted. The fitting formulas are shown in Equations (9)–(12) respectively.

(9) $$E_{d}{}_{\left(\mathrm{NLS}\right)}=19.42y+112.65,R^{2}=0.93$$

(10) $$E_{d}{}_{\left(\mathrm{NFLS},N=6\%\right)}=-14.27x+207.77,R^{2}=0.94$$

(11) $$E_{d}{}_{\left(\mathrm{FLS}\right)}=-6.91x+138.85,R^{2}=0.81$$

(12) $$E_{d}{}_{\left(\mathrm{NFLS},F=0.75\%\right)}=19.66y^{2}-86.71y+180.63,R^{2}=0.98$$

where *E_d_* is dynamic elastic modulus (MPa).

#### 3.2.3. Relationship between Static and Dynamic Elastic Modulus

**Figure 11:**
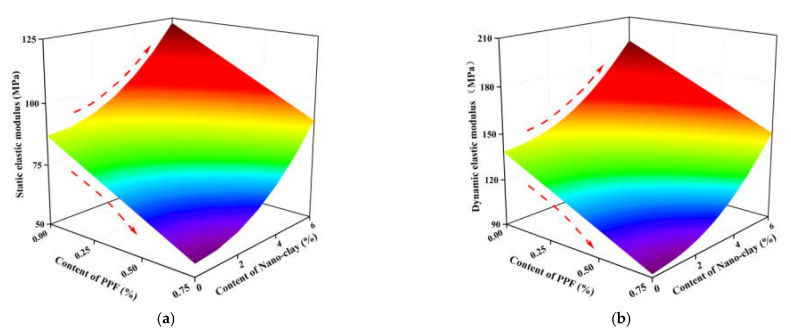
Influence of PPF and nano clay content variation on elastic modulus prediction model: (**a**) static elastic modulus; (**b**) dynamic elastic modulus.

Comparing Figure 9 and Figure 10, it can be found that the content of polypropylene fiber and nano clay has a significant impact on the static and dynamic elastic modulus of NFLS. Therefore, the prediction model of the impact of different content of polypropylene fiber and nano clay on the static and dynamic elastic modulus of modified LS can be established. Figure 11a,b shows the prediction model of the relationship between the content of polypropylene fiber, nano clay, and static and dynamic elastic modulus. It reflects the relationship between the static and dynamic elastic modulus of NFLS and the content of polypropylene fiber and nano clay. Under the same nano clay content, the predicted model surface shows a downward convex shape with the increase of polypropylene fiber content, indicating that the static and dynamic elastic modulus of NFLS first decreases and then increases with the change of polypropylene fiber content. Under the same polypropylene fiber content, the prediction model surface increases with the increase of nano clay content, indicating that the static and dynamic elastic modulus of NFLS increases linearly with the increase of nano clay content. The fitting formulas of static and dynamic elastic modulus of NFLS are shown in Equations (13) and (14):

(13) $$E=86.9-42.39x+0.99y^{2},R^{2}=0.92$$

(14) $$E_{d}=138.71-61.699x+1.526y^{2},R^{2}=0.85$$

**Figure 12:**
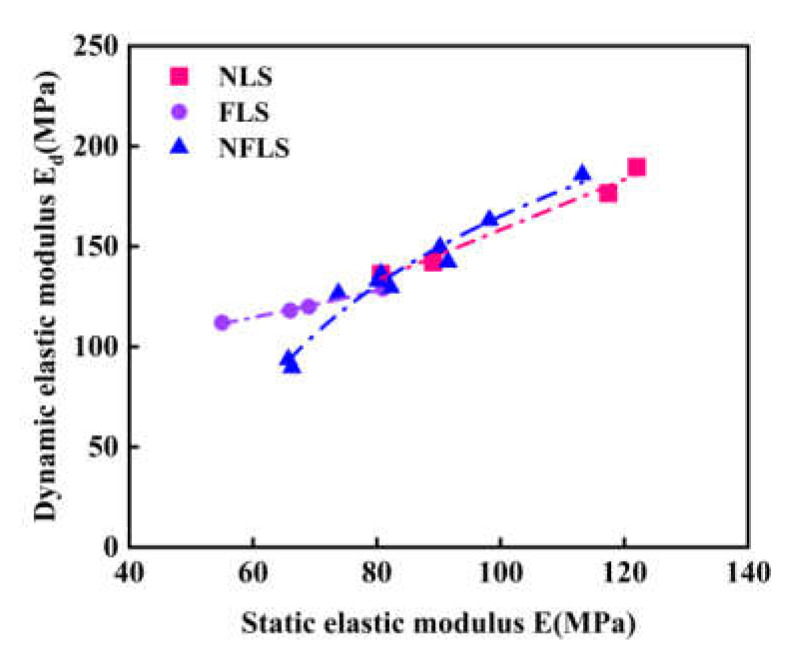
Relationship between static and dynamic elastic modulus.

From the comparison of static and dynamic elastic modulus prediction models according to Figure 11, it can be found that the change trend between the static and dynamic elastic modulus of NFLS and the content of nano clay and polypropylene fiber is basically the same. In the actual road engineering construction, the static elastic modulus is easy to obtain, but in the complex engineering environment, it is difficult to obtain the dynamic elastic modulus through the precise dynamic triaxial test. The static and dynamic elastic modulus test data of NFLS are fitted to establish the variation relationship of them, as shown in Figure 12. The static and dynamic elastic modulus of NLS, FLS, and NFLS conforms to the linear, exponential, and logarithmic relations respectively, and the fitting formula is shown in Equations (15)–(17).

(15) $$E_{d}{}_{\left(\mathrm{NLS}\right)}=0.6557E+75.325,R^{2}=0.99$$

(16) $$E_{d}{}_{\left(\mathrm{FLS}\right)}=29.382e^{0.0208E},R^{2}=0.95$$

(17) $$E_{d}{}_{\left(\mathrm{NFLS}\right)}=165.66\ln\left(E\right)-597.22,R^{2}=0.96$$

### 3.3. Stress–Strain Damage Model and Damage Evolution Law of NFLS

According to the UCS test data, the elastic modulus of NFLS is substituted into the objective function [25,26], and the parameters *λ* and *ζ* are identified by particle swarm optimization algorithm. The identification results are shown in Table 4. The mean value of damage variables *μ_D_* is calculated according to the identification results, and the stress–strain random damage model is established by using *λ* and *ζ*.

**Figure 13:**
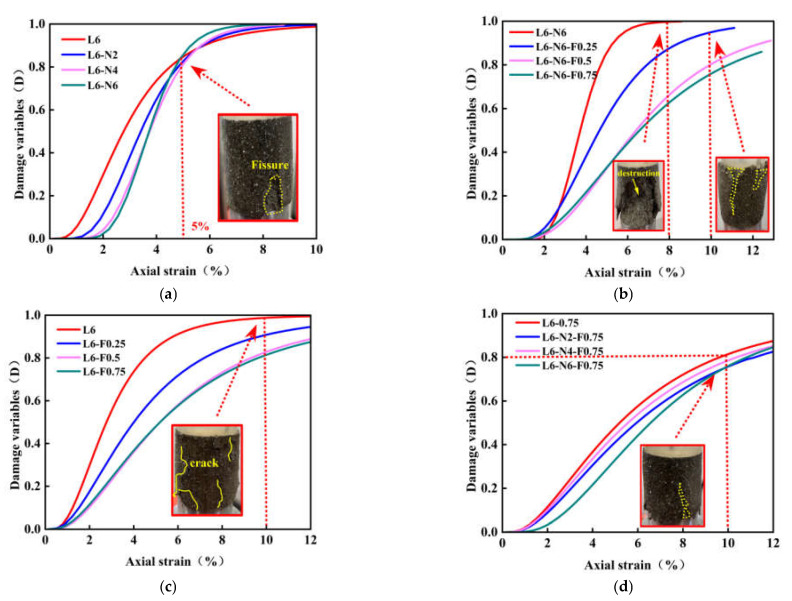
Mesoscopic damage evolution: (**a**) change of NLS with nano clay content; (**b**) changes of NFLS (N = 6%) with PPF content; (**c**) changes of FLS with PPF content; (**d**) change of NFLS (F = 0.75%) with nano clay content.

Figure 13a–d shows the damage evolution curves of NFLS. When *D* = 0, the sample is not damaged. When *D* = 1, the sample is completely destroyed. When the strain is less than 1%, because the last day of curing is immersion curing, the early stage is mainly the compression process of sample pores, and there is no damage. With the increase of strain, the specimen is gradually compressed and destroyed until the damage variable *D* = 1 and the specimen is completely destroyed.

Figure 13a shows that in the early stage of NLS test, before the strain reaches 1%, the sample is in the compaction stage, and the stress changes little with the strain. When the strain increases from 1% to 5%, the specimen is mainly in the linear elastic stage and plastic stage, the stress changes obviously with the strain, and the damage accumulates gradually. When the strain is 5%, the damage variable *D* = 0.8, the sample begins to crack and plastic failure. When the strain is greater than 5%, the damage variable *D* tends to be stable with the increase of strain until the specimen is completely destroyed. Figure 13b shows the change of damage variable *D* of NFLS with the content of polypropylene fiber when the content of nano clay is 6%. When the strain is 8%, the damage variable of NFLS without fiber is *D* = 1 and the sample is completely destroyed. Then, with the increase of fiber content, the destruction speed of the sample slows down. Figure 13c reflects the change of damage variable *D* of FLS with polypropylene content. When the strain of the sample without fiber is 10%, the damage variable *D* = 1, and the sample is completely destroyed. With the increase of the content of polypropylene fiber, the failure rate of FLS slows down, and the sample is not completely destroyed until the strain is 12%. Figure 13d shows the curves of the damage variable *D* of NFLS with the content of nano clay when the content of fiber is fixed at 0.75%. With the increase of nano clay content, the damage curve is dense and the plastic damage is obvious. Due to the addition of polypropylene fiber, the damage of the sample slows down. When the strain is 10%, the sample is not completely damaged. On the one hand, nano clay can fill the pores between soil particles and delay the development of cracks. On the other hand, the fiber plays a reinforcing role, provides tension, and inhibits its plastic deformation.

## 4. Conclusions

The elastic modulus and damage stress–strain model of NFLS are studied through UCS and dynamic triaxial test, and the following conclusions can be obtained.

(1) Polypropylene fiber and nano clay can significantly modify the strength of LS. The growth rate of UCS and residual strength of NFLS is the most significant when the content of polypropylene fiber is 0.75% and the content of nano clay is 6%. On the one hand, nano clay can promote the reaction between lime and soil, and on the other hand, it can react with LS to produce cementitious materials such as hydrated calcium silicate and hydrated calcium aluminate. Nano clay and the generated cementitious material can fill the pores between fiber and soil, enhance the interfacial friction between fiber and soil, and improve the strength of NFLS. UCS can be increased by up to 103%. Polypropylene fiber enhances the ductility of NFLS, slows down the development of cracks, and improves its residual strength. The residual strength can be increased by 827%.
(2) The static and dynamic elastic modulus of NLS, FLS, and NFLS are in functional relationship with the content of polypropylene fiber and nano clay. The addition of nano clay can improve the static and dynamic elastic modulus of NFLS and enhance the ability to resist deformation. In addition, the static and dynamic elastic modulus of NLS, FLS, and NFLS conform to linear, exponential, and logarithmic relationships respectively.
(3) The meso random damage model can characterize the stress–strain relationship of NFLS under axial load. At the same time, the damage variable *D* can further explain the relationship between strain and NFLS damage and failure, and reflect the damage and failure of soil. When the strain is 10%, the rising trend of damage variable *D* of NFLS slows down with the increase of polypropylene fiber and nano clay content, and the damage variable *D* decreases with the increase of nano clay content, indicating that nano clay can improve the strength of NFLS. When the damage variable *D* is 0.8, the axial strain of NFLS increases with the increase of the content of polypropylene fiber and nano clay, and the axial strain increases from 8% to 12% with the increase of the content of polypropylene fiber, indicating that polypropylene fiber can improve the ductility of NFLS. Adding polypropylene fiber and nano clay can effectively improve the damage resistance of NFLS.

The UCS and residual strength of NFLS can be significantly improved by modifying LS with nano clay and polypropylene fiber, and the brittle failure mode of lime soil can be improved. Subsequent microscopic tests can also be carried out to further explain the reasons for the enhancement of NFLS mechanical properties from the perspective of chemical mechanism, providing a theoretical basis for the practical application of NFLS in road engineering.

## Figures and Tables

**Table 1:** Physical properties of soil.

Natural Moisture Content (%)	Specific Gravity	Uniformity Coefficient Cu	Curvature Coefficient Cc	Liquid Limit (%)	Plastic Limit (%)	Plasticity Index (%)
33	2.53	12.2	1.24	37.8	17.2	20.6

**Table 2:** Mix ratio design.

NO	Lime Content (%)	Nano Clay Content (%)	PP Fiber Content (%)	Maximum Dry Density (g/cm^3^)	Optimum Moisture Content (%)	Compactness (%)	Curing Time (d)
LS	6	0	0	1.762	17.5	98	7
NLS	6	2, 4, 6	0				
FLS	6	0	0.25, 0.5, 0.75				
NFLS	6	2, 4, 6	0.25, 0.5, 0.75				

Note, L, N, and F respectively represent lime, nano clay, and polypropylene fiber. Lime content, nano clay content, and polypropylene content are the percentage of dry soil mass, and water content is the percentage of all dry mixtures.

**Table 3:** Test results of UCS test.

NO	UCS (kPa)	Residual Strength (kPa)	Elastic Modulus (MPa)
L6	1239	192	81
L6-N2	1425	321	89
L6-N4	1635	301	117
L6-N6	1947	246	122
L6-F0.25	1439	381	66
L6-F0.5	1913	1228	69
L6-F0.75	1794	1315	55
L6-N2-F0.75	2037	1498	66
L6-N4-F0.75	2057	1596	65
L6-N6-F0.25	2007	840	113
L6-N6-F0.5	2193	1245	98
L6-N6-F0.75	2513	1762	90

**Table 4:** Calculation results of *λ* and *ζ*.

NO	*λ*	*ζ*	*η* (%)
L6	−3.5443	0.6054	1.0
L6-N2	−3.2778	0.4326	1.9
L6-N4	−3.1601	0.3403	2.5
L6-N6	−3.1448	0.2955	1.9
L6-F0.25	−3.2111	0.7107	3.3
L6-F0.5	−2.9546	0.7141	2.7
L6-F0.75	−2.8662	0.7064	3.6
L6-N2-F0.75	−2.8381	0.7826	0.5
L6-N4-F0.75	−2.9024	0.7730	1.5
L6-N6-F0.25	−2.9897	0.5031	1.6
L6-N6-F0.5	−2.6872	0.5547	1.3
L6-N6-F0.75	−2.6819	0.6176	0.8

Note, *η* = *q*/UCS, $q=\frac{\sqrt{\sum_{i}^{m}\left(\sigma_{i}-\sigma (\varepsilon_{i})\right)}}{m}$, where *σ_i_* and *σ* (*ε_i_*) is the measured stress and model calculated stress with the strain is *ε_i_*, respectively, and m is the number of measured data groups.

## Data Availability

Not applicable.
